# Alcohol consumption and labour market participation: a prospective cohort study of transitions between work, unemployment, sickness absence, and social benefits

**DOI:** 10.1007/s10654-018-0476-7

**Published:** 2019-01-10

**Authors:** Maja Bæksgaard Jørgensen, Jacob Pedersen, Lau Caspar Thygesen, Cathrine Juel Lau, Anne Illemann Christensen, Ulrik Becker, Janne S. Tolstrup

**Affiliations:** 1 National Institute of Public Health, University of Southern Denmark, Studiestræde 6, 1455 Copenhagen, Denmark; 2 The National Research Centre for the Working Environment, Lersø Parkallé 105, 2100 Copenhagen, Denmark; 3 Center for Clinical Research and Prevention, Bispebjerg and Frederiksberg Hospital, The Capital Region, Copenhagen, Denmark; 4 Gastrounit, Medical Division, Copenhagen University Hospital Hvidovre, 2650 Hvidovre, Denmark

**Keywords:** Alcohol, Labour market participation, Prospective cohort study

## Abstract

The aim of this study was to investigate the association of alcohol consumption and problem drinking on transitions between work, unemployment, sickness absence and social benefits. Participants were 86,417 men and women aged 18–60 years who participated in the Danish National Health Survey in 2010. Information on alcohol consumption (units per week) and problem drinking (CAGE-C score of 4–6) was obtained by questionnaire. The primary outcome was labour market attachment. Information on labour market attachment was obtained from the national administrative registers during a 5-year follow-up period. Using Cox proportional hazards models, we estimated hazard ratios (HR) for transitions between work, unemployment, sickness absence and social benefits. Analyses were adjusted for potential confounders associated with demography, health, and socio-economy. High alcohol consumption and problem drinking was associated with higher probability of unemployment, sickness absence and social benefits among participants employed at baseline compared with participants who consumed 1–6 drinks/week. High alcohol consumption and problem drinking was associated with lower probability of returning to work among participants receiving sickness absence at baseline compared with participants who consumed 1–6 drinks/week and with non-problem drinkers: HRs were 0.75 (0.58–0.98) for 35+ drinks per week and 0.81 (0.65–1.00) for problem drinking (CAGE-C score of 4–6). Similar trends for weekly alcohol consumption and problem drinking were observed among participants who were unemployed at baseline. In summary, problem drinking has adverse consequences for labour market participation and is associated with higher probability of losing a job and a lower chance of becoming employed again.

**Electronic supplementary material:**

The online version of this article (10.1007/s10654-018-0476-7) contains supplementary material, which is available to authorized users.

## Introduction

For most people, work is the main source of financial support and ensures that basic needs, such as being able to maintain a home and support a family, are met. Work also has other roles, for example it provides social identity and a feeling of self-worth. Employment status and lifestyle as well as mental and physical health are intimately connected and losing one’s job is associated with increased risk of depression [1, 2], cardiovascular disease [3–12], and all-cause mortality [5, 13–16]. Additionally, becoming unemployed is associated with the initiation or extension of unhealthy habits such as smoking and hazardous drinking [17, 18]. Hence, knowledge of risk factors for job loss and barriers for getting back to work is a great public health concern.

Alcohol is linked to more than 200 diseases and injuries and is the fifth leading risk factor for disease, disability and death worldwide [19, 20]. Harmful alcohol consumption places a vast burden on healthcare and social systems, and in high-and middle-income countries, the cost of this abuse is significant and corresponds to approximately > 1% of the Gross National Product (GNP) [21]. While associations between alcohol and health outcomes have been extensively studied, less is known about the impact of alcohol on labour market transitions such as becoming unemployed or getting back to work after being unemployed—i.e., downward social selection. Some studies indicate that heavy drinking and alcohol problems are associated with an increased risk of being laid off [22, 23] but associations between the full spectrum of alcohol consumption and risk of unemployment are unknown, as is the impact of alcohol on the probability of getting back to work.

In this study, we aim to investigate the association of weekly alcohol consumption and problem drinking on the transition between different labour market endpoints (i.e. work, unemployment, sickness absence and social benefits) in order to identify effective prevention strategies and reduce exclusion within society. We contribute to the literature by using a large, population-based cohort linked to valid administrative registers. Participants were followed for up to 5 years without loss to follow-up while adjustments were made to take account of socio-economic, demographic, and health-related factors were adjusted.

## Methods

This study is based on The Danish National Health Survey 2010 (DNHS-2010) linked to national registers using the unique personal identification number given to all residents in Denmark.

### Study design and participants

The DNHS-2010 is a national health survey conducted in Denmark. DNHS-2010 was based on six mutually exclusive random samples: a national sample and one in each of the five regions of Denmark (the Capital Region of Denmark, Region Zealand, the Region of Southern Denmark, the Central Denmark Region, the North Denmark Region). The sample was randomly selected from the adult population in Denmark, aged 16 years or older, using the Danish Civil Registration System [24]. The sample included 298,850 individuals alive and living in Denmark on 1 January 2010. All selected individuals received a letter that described the purpose of the study and emphasized that participation was voluntary. The letter invited individuals to either fill in the enclosed paper questionnaire or to complete a web questionnaire. Data was collected from February to April 2010. In case of non-response, two postal reminders were sent. The questionnaire was completed by 177,639 individuals, which corresponds to 59.5% of the total sample. A detailed description of the sampling procedure and design has been published elsewhere [25].

#### Assessment of alcohol consumption

Participants were asked how many alcoholic drinks (beer, cider, wine, drinks and spirits) they consumed on each weekday in a typical week. A drink was defined as one beer, one shot or one glass of wine, which corresponded to approximately 12 g of pure alcohol. The number of drinks per week was calculated and divided into five response categories: no intake, 1–6 drinks/week, 7–13, 14–20, 21–34, and ≥ 35 drinks/week. The cut-off values were in line with the Danish guidelines for sensible drinking proposed by the Danish Health Authority [26, 27]. Problem drinking was measured by the CAGE-C questionnaire (Cut down, Annoyed, Guilty, Early-morning—Copenhagen) [28], and this study included six items from the questionnaire: (1) Have you, within the past year, felt that you should cut down on your drinking? (2) Have people, within the past year, annoyed you by criticizing your drinking? (3) Have you, within the past year, felt bad or guilty about your drinking? (4) Have you, within the past year, had a drink first thing in the morning to steady your nerves or to get rid of a hangover from time to time? (5) How many days per week do you drink alcohol (positive answer = 4 days or more)? (6) Do you drink alcohol on weekdays outside mealtimes? The CAGE-C was divided into three categories (no positive answers, 1–3 positive answers, and 4–6 positive answers on the CAGE-C questionnaire) and 4–6 positive answers was defined as problem drinking.

#### Assessment of labour market participation

In Denmark, the labour market is defined by a flexicurity system that includes a high degree of economic compensation and security from the state of Denmark in case of exclusion from the labour market, for example due to unemployment or reduced work ability (security), but is characterized at the same time by a high turnover of jobs and an active (e.g. flexible) labour market policy [29, 30]. Data on labour market participation was obtained from DREAM (The Danish Register-based Evaluation of Marginalization). DREAM contains weekly information for every Danish citizen or resident who receives social transfer payments. The database has been shown to be valid for follow-up of the economic and social consequences of disease [31] and is regularly updated [32]. Labour market outcomes were divided into four categories: work, unemployment, sickness absence, and other social benefit. Selected codes of social transfer payments are available in Table I in the supplementary material. The ‘work’ state contains all periods during which participants were not receiving any social transfer payments. Social transfer payments obtained from DREAM were divided into three endpoints: unemployment (available to the labour market including on unemployment benefit and social cash-benefits), sickness absence (temporary unavailability to labour market due to health problems), and social benefits (temporary unavailability to labour market). The weekly information was transformed to start and end dates, and we obtained data from Week 5 of 2010 (time of baseline) until Week 5 of 2015. Information on emigration and vital status was obtained from the Danish Civil Registration System [33].

#### Assessment of potential confounders

Analyses were adjusted for a priori identified potential confounders known to be associated with both alcohol consumption and labour market outcomes [34, 35]. Information on confounders was obtained in the year of baseline. Three types of confounders were included: demographic, health-related, and socio-economic factors. *Demographic confounders*: Registers from Statistics Denmark [36] were used to gain information on sex, age, cohabitation status (living alone versus not) and geography (five regions). *Health*-*related confounders:* The Charlson comorbidity index was calculated from the National Patient Register [37], defined according to 19 selected diseases [38, 39] and categorized in two levels (none and any kind of comorbidity). Mental illness and disorders and smoking habits were obtained from the DNHS-2010 questionnaire. Participants reported if they had mental illness and disorders (no, yes—now or previously with current repercussions) and if they were smokers, ex-smokers or current smokers and current smokers reported the number of cigarettes smoked daily. Smoking was categorized in five groups: never smoked, ex-smoker, non-cigarette smoker, 1–14, and 15+ cigarettes/day. *Socio*-*economic confounders:* Information on length of education was obtained from Danish education registers [40] and was categorized as less than 10 years, 10–12 years, and more than 12 years of education, which corresponded to primary, upper secondary or vocational and higher education, respectively. Moreover, to adjust for labour market attachment, four factors were included holding information on employment status the year prior to baseline, i.e. amount (%) of time working, receiving unemployed benefit, sickness absence, and social benefit.

#### Final study population

**Fig. 1:**
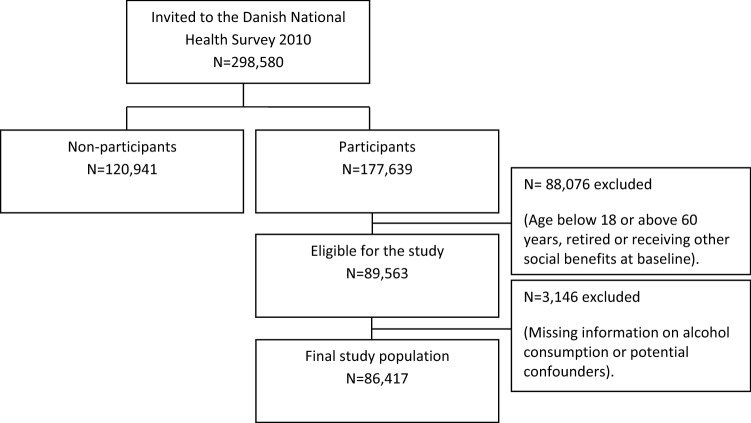
Chart showing the flow from the Danish National Health Survey (DNHS) 2010 to the final study population

Analyses were restricted to participants aged 18–60 years who were working, in receipt of unemployment or sickness absence, and had intact data sets on alcohol consumption or potential confounders, with the result that 86,417 individuals were eligible for analysis (Fig. 1).

### Statistical analysis

Baseline characteristics of participants were described with median values (5th–95th percentiles) for continuous variables and proportions for categorical variables. All participants were followed from baseline (1 February 2010), when the questionnaires were distributed, to the date of labour market outcomes (mutually exclusive outcomes were included—see Table 2), death, emigration, age limit of 65 years, or the end of follow-up (31 January 2015), whichever came first.

Figure 5 gives a visual illustration of the 12 possible transitions (work, unemployment, sickness absence, and social benefits); however, we did not have sufficient statistical power to study transitions from sickness absence to social benefits and receiving social benefits and so, consequently, a total of eight transitions were analyzed. Each transition was analyzed separately using the Cox Proportional Hazard Model with time since baseline (in days) as the underlying time scale. Reference categories were low alcohol consumption (1–6 drinks per week) or non-problem drinking (4–6 positive answers in the CAGE-C questionnaire). Analyses were adjusted for age, gender, cohabitation status, educational level, Charlson comorbidity index [37], smoking habits, geographic region and labour market participation in the year prior to baseline. The proportional hazards assumption in the Cox model was evaluated by visual inspection of log–log plots with no violations detected. Tests for linear and quadratic trends were conducted by treating the median and squared median alcohol consumption within categories as continuous variables. SAS version 9.4 and STATA version 15 were used for the statistical analyses. In all tests, *p* values < 5% were considered statistically significant.

### Sensitivity analyses

We conducted sensitivity analyses using the Fine-Gray competing risk regression model. However, including competing risks had little impact on results (supplementary materials figures I–III).

## Results

Of the 86,417 participants, 40,912 (47%) were men (Table 1). The median alcohol consumption was 7.6 (5th–95th, 0–24), 10.0 (5th–95th, 0–36) and 8.3 (5th–95th, 0–30) drinks per week among participants who were working, unemployed and receiving sickness absence. In general, the group of unemployed participants had a shorter education (< 10 years) and a more adverse risk factor profile (including problem drinking, current smoker and living alone). However, participants receiving sickness absence had the largest proportion of comorbidity and mental illness and disorders compared with participants who were working or unemployed.

**Table 1:** Baseline characteristics of 86,417 participating in the Danish National Health Survey 2010 according to income type

	Types of income			
	All	Working (n = 77,746, 90.0%)	Unemployed (n = 5569, 6.4%)	Sickness absence (3102, 3.6%)
Men (n, %)	40,912 (47.3)	36,547 (47.0)	3182 (57.1)	1183 (38.1)
Age, median yrs (5th–95th)	43.4 (23–58)	43.5 (23–58)	42.3 (23–59)	45.5 (25–59)
Alcohol, drinks/wk^a^, median (5th–95th)	7.7 (0–25)	7.6 (0–24)	10.0 (0–36)	8.3 (0–30)
Problem drinking^b^ (n, %)	2588 (3.0)	2159 (2.8)	286 (5.1)	143 (4.6)
Primary school education^c^ (n, %)	16,695 (19.3)	13,994 (18.0)	1846 (33.2)	855 (27.6)
Upper secondary/vocational education^d^ (n, %)	38,352 (44.4)	34,307 (44.1)	2606 (46.8)	1439 (46.4)
Higher education^e^ (n, %)	31,370 (36.3)	29,445 (37.8)	1117 (20.1)	808 (26.1)
Current smoker (n, %)	21,037 (24.3)	17,889 (23.0)	2127 (38.1)	1021 (32.9)
Living alone (n, %)	17,812 (20.6)	15,228 (19.6)	1808 (32.4)	782 (25.1)
Charlson comorbidity index ≥ 1 (n, %)	4364 (5.1)	3543 (4.6)	305 (5.5)	516 (16.6)
Mental illness and disorders (n, %)	1289 (1.5)	849 (1.10)	150 (2.7)	290 (9.3)

Data are presented as median (5th percentile, 95 th percentile) or n (%)

^a^One drink corresponds to 12 g of pure alcohol

^b^Problem drinking was defined as a CAGE-C score of 4–6

^c^< 10 years of education

^d^10–12 years of education

^e^> 12 years of education

### Transitions from work to unemployment, sickness absence, and social benefit

The mean follow-up time varied between the types of labour market participation. At the end of follow-up, a total of 9499 participants had transferred to unemployment, 17,585 to sickness absence and 686 to social benefits among participants working at baseline (n = 77,746) (Table 2).

**Table 2:** Number (%) and mean follow-up time (5th–95th percentiles, in years) for transitions between work, unemployment and sickness absence at baseline to work, unemployment, sickness absence and social benefits at follow up, in 86,417 participants from the Danish National Health Survey 2010

	Types of labour market participation				
	To work	To unemployment	To sickness absence	To social benefits	To censoring
From work 77,746 (90.0)	–	9499 (12.2) 1.8 years (0.06–4.56)	17,585 (22.6) 1.9 years (0.08–4.62)	686 (0.9) 1.5 years (0.04–4.16)	49,976 (64.3) 4.8 (3.41–5.0)
From unemployment 5569 (6.4)	4517 (81.1) 0.4 years (0.04–1.34)	–	767 (13.7) 0.5 years (0.02–1.76)	182 (3.3) 0.5 years (0.02–1.49)	103 (1.9) 0.6 years (0.04–2.17)
From sickness absence 3102 (3.6)	2302 (74.2) 0.4 years (0.02–1.42)	442 (14.3) 0.4 years (0.02–1.42)	–	71 (2.3) 1.1 years (0.08–2.72)	287 (9.2) 0.9 years (0.15–1.99)

**Fig. 2:**
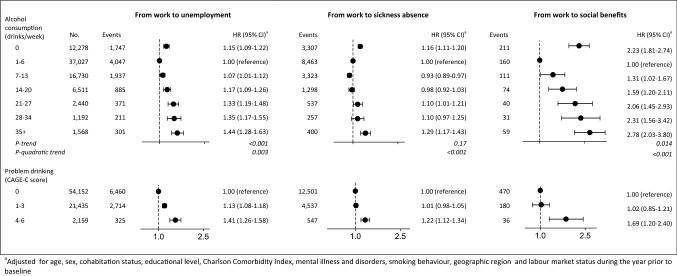
Hazard ratios and 95% confidence intervals for transitions from work to unemployment, sickness absence or social benefits according to weekly alcohol consumption and problem drinking in 77,746 participating in the Danish National Health Survey 2010

Among participants who were working at baseline, hazard ratios (HR) of unemployment, sickness absence and social benefit were higher among abstainers and participants with a high alcohol consumption than participants with low consumption (1–6 drinks per week) (Fig. 2). The HR of unemployment were 1.15 (95% CI 1.09–1.22) for 0 drinks per week, 1.07 (95% CI 1.01–1.12) for 7–13 drinks per week, 1.17 (95% CI 1.09–1.26) for 14–20 drinks per week, 1.33 (95% CI 1.19–1.48) for 21–27 drinks per week, 1.35 (95% CI 1.17–1.55) for 28–34 drinks per week, and 1.44 (95% CI 1.28–1.63) for 35+ drinks per week. For the transition between work and sickness absence, the HR was lower among those who consumed 7–13 drinks per week with an HR of 0.93 (95% CI 0.89–0.97).

### Transitions from unemployment to work, sickness absence and other social benefits

**Fig. 3:**
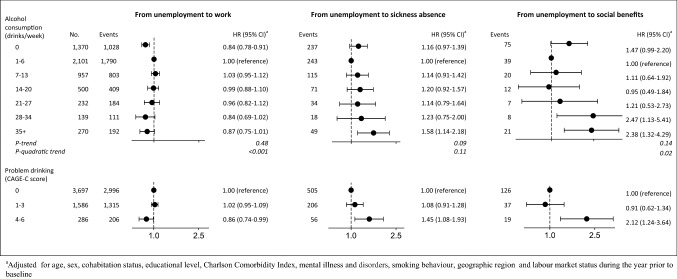
Hazard ratios and 95% confidence intervals for transitions from work unemployment to work, sickness absence and social benefit by weekly alcohol consumption and CAGE-C in 5569 participating in the Danish National Health Survey 2010

Among participants unemployed at baseline (n = 5569), a total of 4517 participants had returned to work, 767 had transferred to sickness absence and 182 had transferred to social benefits at the end of follow-up (Table 2). Heavy alcohol consumption of 35+ drinks per week was associated with a higher risk of transfer to sickness absence among participants who were unemployed at baseline. When compared with participants who drank 1–6 drinks per week, the HR among drinkers of 35+ drinks per week was 1.58 (95% CI 1.14–2.18). Additionally, the HR of transfer from unemployment to social benefit was also higher among participants with high alcohol consumption of 28+ drinks per week when compared with participants who consumed 1–6 drinks per week (Fig. 3).

### Transitions from sickness absence to work and unemployment

**Fig. 4:**
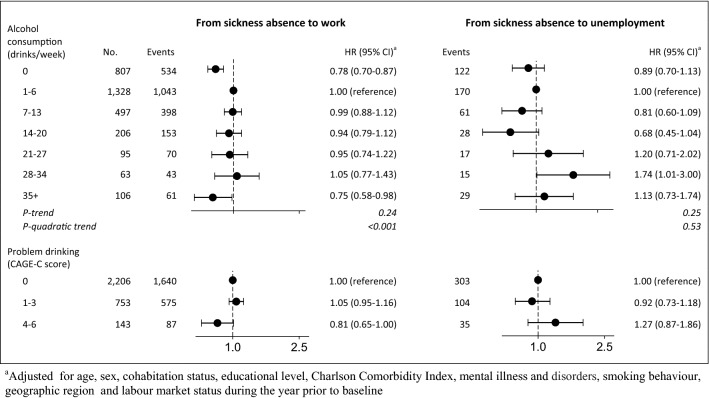
Hazard ratios and 95% confidence intervals for transitions from sickness absence to work, unemployment and social benefit by weekly alcohol consumption in 3102 participating in the Danish National Health Survey 2010

A total of 2302 participants had returned to work and 442 transferred to unemployment (Table 2). Among participants receiving sickness absence at baseline, we found a lower HR of returning to work among abstainers and consumers of 35+ drinks per week with an HR of 0.78 (95% CI 0.70–0.87) and 0.75 (95% CI 0.58–0.98) when compared with those who had 1–6 drinks per week. For the transition between sickness absence and unemployment, the HR was higher among consumers of 28–34 drinks per week with an HR of 1.74 (95% CI 1.01–3.00) (Fig. 4).

### Problem drinking and transitions between work, employment, sickness absence and social benefits

**Fig. 5:**
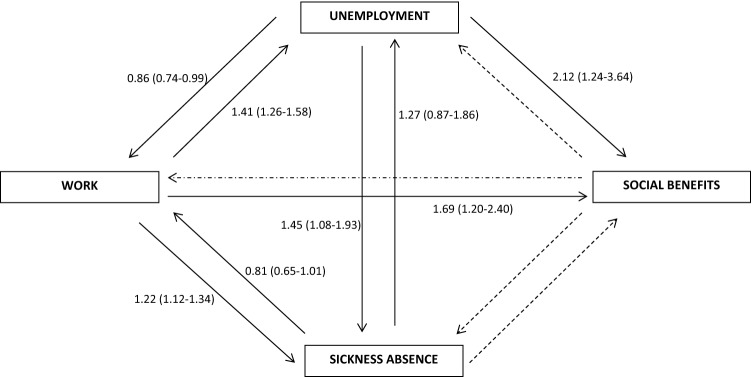
Hazard ratios and 95% confidence intervals for transitions between work, unemployment and sickness absence at baseline to work, unemployment, sickness absence and social benefits at follow-up according to problem drinking (CAGE-C score of 4–6). Reference group was no problem drinking. Dashed arrows represent transitions that was not possible to analyze, due to low power. The eight transitions were adjusted for age, gender, cohabitation status, educational level, Charlson Comorbidity Index, mental illness and disorders, smoking behaviour, geographic region and labour market status during the year prior to baseline

Figure 5 shows the HR for transitions between all four endpoints according to problem drinking (CAGE-C score of 4–6). Problem drinking was associated with a higher HR of unemployment, sickness absence and social benefits among participants who were working at baseline with an HR of 1.41 (95% CI 1.26–1.58), 1.22 (95% CI 1.12–1.34), and 1.69 (95% CI 1.20–2.40) when compared with non-problem drinkers.

A significantly lower probability of returning to work was observed among participants who were problem drinkers and unemployed at baseline compared with non-problem drinkers with an HR of 0.86 (95% CI 0.74–0.99). Furthermore, problem drinking was associated with a higher HR of transfer to sickness absence and social benefits among participants who were unemployed at baseline compared with non-problem drinkers.

## Discussion

Results from this study showed that high amounts of weekly alcohol consumption and problem drinking were associated with an increased HR of unemployment, sickness absence and social benefits among participants employed at baseline. Moreover, results showed that high amounts of weekly alcohol consumption and problem drinking were associated with lower probability of returning to work after being unemployed or receiving sickness absence and increased the HR of transfer to social benefits (sickness absence and social benefits). Analyses of all eight transitions showed that abstainers had a similar HR to individuals with heavy alcohol consumption.

### Strengths and limitations

The main strengths of this prospective cohort study are the large study sample and virtually complete follow-up for labour market outcomes through linkage to nationwide registers. A further strength is the ability to examine labour market participants across a wide range of alcohol consumption and problem drinking.

Several limitations, however, are evident in this study. Firstly, measures of alcohol consumption were self-reported and likely to be underestimated [41]. Even so, the methods applied in this study are generally regarded to be valid and found suitable for use in epidemiological studies [42] as results are also similar using different methods of estimating alcohol use. Secondly, we were unable to separate abstainers and former drinkers due to limitations in the DNHS-2010 questionnaire. This information would have been valuable as individuals may have altered their weekly alcohol consumption in response to health conditions and this might explain the similar risk estimates among abstainers and heavy drinkers [43–45]. This phenomenon is often referred to as the ‘sick quitter’ effect or former drinker’s bias [46, 47] and as a consequence, all analyses were adjusted for comorbidities (the Charlson comorbidity index) and mental illness and disorders to account for comorbidity. However, this issue was not a problem when analyzing problem drinking. Here the lowest risk estimates belong to individuals categorized as non-problem drinkers (no positive answers on the CAGE-C questionnare).

Thirdly, we used the DREAM register to obtain information on labour market participants. It is important to note that invisible unemployment could occur (e.g. if an unemployed individual is not in receipt of any social transfer benefit) [48]. Additionally, no record was made to distinguish between individuals who are receiving sickness benefit but are still employed and individuals who are receiving sickness benefit but are unemployed. This DREAM register has previously been proven suitable for analyses of labour market consequences [32]. Fourthly, although we made adjustments for multiple potential confounders, we cannot rule out the possibility of residual confounding or unmeasured confounding. Factors such as occupational status, income, or other risk determinants might also be associated with exposure and endpoints. A proxy variable for occupational status and income was available through information on education found in the Danish education registers.

Fifthly, the study population was restricted to participants aged 18–60 years, which is a relatively large age range. We conducted sensitivity analyses that excluded participants aged 55–60 years, as the older population could have an increased HR of sickness absence or differ in other ways from the general population. This exclusion did not substantially change the estimates. Sixthly, only 59.5% of those invited participated and the people who declined may have had different risk profiles to those who chose to participate. A recently conducted Danish study compared cause-specific mortality and morbidity among survey respondents and non-respondents from the Danish Health and Morbidity Survey population collected in 2000 and 2005 [49]. This study found that non-respondents in Denmark have an increased hazard ratio of alcohol, drug and smoking-related mortality and morbidity when compared with respondents [49]. This may have affected the results and some caution should be taken when interpreting the findings.

A final limitation is that the interpretation of results may be confounded by unknown or unmeasured factors associated with exposure and endpoint (labour market participation), which could have biased the risk estimates [35]. It is likely that both exposure and endpoint are associated with other physical, social, and behavioural risk factors not included in the analyses, such as impulsivity, lower cognitive ability, or, socioeconomic disadvantages occurring early in life or genes [16, 49–51].We included information on previously applied confounders; [22] but we were unable to apply a life-course perspective because information was not available in this study and confounding due to unknown or unmeasured factors and residual confounding due to broad categorization cannot be excluded. Previous studies suggest that this is only a problem if the confounding factors are strongly associated with endpoints and distributed differently between the exposure groups [52].

### Comparison with other studies

The mechanisms by which alcohol could impact on labour market participants are complex and not entirely understood [22, 53]. In the literature it is suggested that moderate alcohol consumption has a beneficial effect on labour outcomes [43, 44, 54–57]; however, these findings are often disputed due to the potential of e.g. selection or former drinker bias [53]. Previous literature has also suggested that heavy alcohol consumption may have an adverse effect on labour market participants in terms of loss of productivity and ability to work. [23, 58–63] In the short term, heavy alcohol consumption may have social and legal consequences, lead to hangovers from binge drinking, and increase absenteeism from work. Additionally, heavy alcohol consumption may cause health problems in the long term [23, 64], creating a vicious circle which may affect labour market participation [16].

To the best of our knowledge, no previous studies have investigated the transitions between different labour market outcomes by weekly alcohol consumption and problem drinking as we have done in this study. The strong evidence base for alcohol consequences on disease contrasts with relatively few studies on alcohol consumption and labour market outcomes. Previous studies have been restricted to single labour market outcomes such as employment, working hours, wages and earnings, absenteeism and unemployment [22]. Consistent with our findings, the literature generally suggests that a non-linear association is present (e.g. moderate alcohol consumers have higher wages and earnings compared to abstainers and heavy alcohol users) [22, 23, 43, 44, 54, 55, 57].

Other studies have examined whether alcohol consumption is related to labour market participation and attachment [23]. Some studies indicate that heavy alcohol consumption increases the probability of being unemployed. [23, 58–63] This is in contrast to other studies that could not find a statistically significant relationship between alcohol consumption and labour market participation [64, 65]. The difference between the results may be partly explained by differences in the sampling methods, study population, data quality and design as well as country-specific characteristics. For example, the majority of studies have been conducted in countries that are not comparable to the Danish labour market conditions and welfare system.

### Implications and future research

Harmful alcohol consumption remains a key public health concern. Alcohol-attributable factors play a major part in producing health inequalities. Having a job and good health are intimately connected. Job loss has an adverse effect on health behaviour as well as on mental and physical health—which could create a vicious circle of growing health inequalities. The available evidence sheds light on a hidden consequence of alcohol consumption. By drawing attention to the field, we hope to stimulate more research that will inform policymakers about the magnitude of the problems.

Additional research should elucidate whether observed associations in this study could be reproduced in other countries. In order to prevent adverse consequences from alcohol consumption, more research is needed to investigate the mechanisms behind the underlying causes. More knowledge might help to clarify public health aims, lead to an increased focus and provide guidance on new ways to target individuals within and outside the labour market. It would also be interesting to study the inverse relationship between unemployment and heavy drinking or alcohol problems.

## Conclusion

In this large prospective cohort study of the general Danish population, the results suggest that high alcohol consumption and problem drinking increases the risk of leaving the labour market and is a barrier to re-employment.

## Electronic supplementary material

Below is the link to the electronic supplementary material.

Supplementary material 1 (DOCX 33 kb)
