# Supplementary material for: Alcohol consumption and labour market participation: a prospective cohort study of transitions between work, unemployment, sickness absence, and social benefits
Source: Eur J Epidemiol. 2019 Jan 10;34(4):397–407. doi: 10.1007/s10654-018-0476-7 (PMC6451700; doi:10.1007/s10654-018-0476-7)
Supplement: Supplementary file 1 — Supplementary material 1 (DOCX 33 kb) [file 10654_2018_476_MOESM1_ESM.docx]

**Supplementary material**

| Table I. Selected codes for transfer payments included in the DREAM database | | | |
| --- | --- | --- | --- |
|  | **Categorizing** | **Transfer payment** | **Code*** |
| Available to the labour marked | **Work** | No transfer payments (self-supporting/part of the workforce) | No entry, 511, 121, 122, 123 |
|  | **Unemployment** |  |  |
|  |  | Unemployment benefit ( incl. special education benefit and subsidized employment) | 111-114, 124-126, 211, 213-218, 231, 232, 299, 151, 152, 153 |
|  |  | Social cash-benefit (available) | 130, 131, 133-139, 140, 143-149 |
| Temporary unavailable to the labour marked | **Sickness absence** |  |  |
|  |  | Sickness absence benefit | 870, 873, 874-878, 890, 893-899 |
|  | **Social benefits** | Social cash-benefit (Temporary unavailable) | 720, 723-729, 730, 731, 733-739 |
| Permanent unavailable for to labour marked | **Censored** |  |  |
|  |  | Public retirement pension | 998 |
|  |  | Anticipatory pension scheme | 783 |
|  |  | Early retirement | 621 |
|  |  | Emigrated | 997 |
|  |  | Dead |  |
| **Transfer payments beyond this categorisation is not included in this study* | | | |

| Figure I. Hazard ratios and 95% confidence intervals for transitions from work to unemployment, sickness absence or social benefits according to weekly alcohol consumption and problem drinking in 77,746 participating in the Danish National Health Survey 2010. | | | | | | | | | | |
| --- | --- | --- | --- | --- | --- | --- | --- | --- | --- | --- |
| Alcohol consumption  (drinks/week) | |  | **From work to unemployment** | |  | **From work to sickness absence** | |  | **From work to social benefits** | |
|  |  | No. | Events | HR (95% CI)^a^ |  | Events | HR (95% CI)^a^ |  | Events | HR (95% CI)^a^ |
|  | 0 | 12,278 | 1,747 | 1.12 (1.05-1.18) |  | 3,307 | 1.13 (1.09-1.18) |  | 211 | 2.17 (1.76-2.69) |
|  | 1-6 | 37,027 | 4,047 | 1.00 (reference) |  | 8,463 | 1.00 (reference) |  | 160 | 1.00 (reference) |
|  | 7-13 | 16,730 | 1,937 | 1.07 (1.01-1.13) |  | 3,323 | 0.92 (0.88-0.96) |  | 111 | 1.30 (1.02-1.66) |
|  | 14-20 | 6,511 | 885 | 1.17 (1.08-1.26) |  | 1,298 | 0.95 (0.89-1.01) |  | 74 | 1.57 (1.18-2.09) |
|  | 21-27 | 2,440 | 371 | 1.29 (1.16-1.44) |  | 537 | 1.07 (0.98-1.17) |  | 40 | 1.91 (1.32-2.76) |
|  | 28-34 | 1,192 | 211 | 1.33 (1.15-1.55) |  | 257 | 1.04 (0.92-1.18) |  | 31 | 2.14 (1.44-3.18) |
|  | 35+ | 1,568 | 301 | 1.34 (1.18-1.52) |  | 400 | 1.20 (1.07-1.33) |  | 59 | 2.56 (1.84-3.55) |
|  | *P-trend* | |  | *0.00* |  |  | *0*.*17* |  |  | *0*.*01* |
|  | *P-quadratic trend* | |  | *0.00* |  |  | *0.00* |  |  | *0.00* |
|  |  |  |  |  |  |  |  |  |  |  |
| Problem drinking  (CAGE-C score) | | |  |  |  |  |  |  |  |  |
|  | 0 | 54,152 | 6,460 | 1.00 (reference) |  | 12,501 | 1.00 (reference) |  | 470 | 1.00 (reference) |
|  | 1-3 | 21,435 | 2,714 | 1.12 (1.07-1.18) |  | 4,537 | 1.00 (0.96-1.03) |  | 180 | 0.99 (0.83-1.19) |
|  | 4-6 | 2,159 | 325 | 1.32 (1.17-1.49) |  | 547 | 1.17 (1.07-1.28) |  | 36 | 1.51 (1.05-2.18) |
|  | | |  |  |  |  |  |  |  |  |
| ^a^Adjusted for age, sex, cohabitation status, educational level, Charlson Comorbidity Index, mental illness and disorders, smoking behaviour, geographic region and labour market status during the year prior to baseline | | | | | | | | | | |

| Figure II. Hazard ratios and 95% confidence intervals for transitions from work unemployment to work, sickness absence and social benefit by weekly alcohol consumption and CAGE-C in 5,569 participating in the Danish National Health Survey 2010. | | | | | | | | | | |
| --- | --- | --- | --- | --- | --- | --- | --- | --- | --- | --- |
| Alcohol consumption  (drinks/week) | |  | **From unemployment to work** | |  | **From unemployment to sickness absence** | |  | **From unemployment to social benefits** | |
|  |  | No. | Events | HR (95% CI)^a^ |  | Events | HR (95% CI)^a^ |  | Events | HR (95% CI)^a^ |
|  | 0 | 1,370 | 1,028 | 0.83 (0.77-0.90) |  | 237 | 1.30 (1.08-1.56) |  | 75 | 1.57 (1.05-2.34) |
|  | 1-6 | 2,101 | 1,790 | 1.00 (reference) |  | 243 | 1.00 (reference) |  | 39 | 1.00 (reference) |
|  | 7-13 | 957 | 803 | 1.00 (0.92-1.08) |  | 115 | 1.11 (0.89-1.39) |  | 20 | 1.03 (0.60-1.75) |
|  | 14-20 | 500 | 409 | 0.94 (0.85-1.06) |  | 71 | 1.30 (0.99-1.70) |  | 12 | 0.83 (0.41-1.70) |
|  | 21-27 | 232 | 184 | 0.89 (0.76-1.04) |  | 34 | 1.36 (0.94-1.95) |  | 7 | 1.09 (0.46-2.57) |
|  | 28-34 | 139 | 111 | 0.80 (0.66-0.96) |  | 18 | 1.23 (0.75-2.02) |  | 8 | 2.43 (1.12-5.25) |
|  | 35+ | 270 | 192 | 0.76 (0.65-0.89) |  | 49 | 1.61 (1.18-2.21) |  | 21 | 2.30 (1.29-4.11) |
|  | *P-trend* | |  | *0.48* |  |  | *0.09* |  |  | *0*.*14* |
|  | *P-quadratic trend* | |  | *0.00* |  |  | *0.11* |  |  | *0*.*02* |
|  |  |  |  |  |  |  |  |  |  |  |
| Problem drinking  (CAGE-C score) | | |  |  |  |  |  |  |  |  |
|  | 0 | 3,697 | 2,996 | 1.00 (reference) |  | 505 | 1.00 (reference) |  | 126 | 1.00 (reference) |
|  | 1-3 | 1,586 | 1,315 | 1.00 (0.94-1.07) |  | 206 | 1.07 (0.91-1.27) |  | 37 | 0.86 (0.59-1.24) |
|  | 4-6 | 286 | 206 | 0.80 (0.69-0.92) |  | 56 | 1.51 (1.15-2.00) |  | 19 | 2.00 (1.19-3.38) |
|  | | |  |  |  |  |  |  |  |  |
| ^a^Adjusted for age, sex, cohabitation status, educational level, Charlson Comorbidity Index, mental illness and disorders, smoking behaviour, geographic region and labour market status during the year prior to baseline | | | | | | | | | | |

| Figure III. Hazard ratios and 95% confidence intervals for transitions from sickness absence to work, unemployment and social benefit by weekly alcohol consumption in 3,102 participating in the Danish National Health Survey 2010. | | | | | | | |
| --- | --- | --- | --- | --- | --- | --- | --- |
| Alcohol consumption  (drinks/week) | |  | **From sickness absence to work** | |  | **From sickness absence to unemployment** | |
|  |  | No. | Events | HR (95% CI)^a^ |  | Events | HR (95% CI)^a^ |
|  | 0 | 807 | 534 | 0.82 (0.74-0.91) |  | 122 | 1.00 (0.78-1.27) |
|  | 1-6 | 1,328 | 1,043 | 1.00 (reference) |  | 170 | 1.00 (reference) |
|  | 7-13 | 497 | 398 | 1.03 (0.93-1.15) |  | 61 | 0.83 (0.61-1.13) |
|  | 14-20 | 206 | 153 | 1.02 (0.86-1.22) |  | 28 | 0.81 (0.51-1.27) |
|  | 21-27 | 95 | 70 | 0.94 (0.73-1.21) |  | 17 | 1.25 (0.77-2.01) |
|  | 28-34 | 63 | 43 | 0.87 (0.62-1.21) |  | 15 | 1.58 (0.94-2.65) |
|  | 35+ | 106 | 61 | 0.81 (0.62-1.05) |  | 29 | 1.15 (0.72-1.83) |
|  | *P-trend* | |  | *0.24* |  |  | *0.25* |
|  | *P-quadratic trend* | |  | *0.00* |  |  | *0.53* |
|  |  |  |  |  |  |  |  |
| Problem drinking  (CAGE-C score) | | |  |  |  |  |  |
|  | 0 | 2,206 | 1,640 | 1.00 (reference) |  | 303 | 1.00 (reference) |
|  | 1-3 | 753 | 575 | 1.06 (0.96-1.17) |  | 104 | 0.90 (0.70-1.15) |
|  | 4-6 | 143 | 87 | 0.86 (0.69-1.07) |  | 35 | 1.29 (0.86-1.94) |
|  | | |  |  |  |  |  |
| ^a^Adjusted for age, sex, cohabitation status, educational level, Charlson Comorbidity Index, mental illness and disorders, smoking behaviour, geographic region and labour market status during the year prior to baseline | | | | | | | |
